# Food processing industry changes across China regions: The case of flour, rice, oil, and other cereal derivative food

**DOI:** 10.1002/fsn3.3190

**Published:** 2022-12-19

**Authors:** Dandan Dou, Fengting Li, Liying Li

**Affiliations:** ^1^ School of Management Henan University of Technology Zhengzhou China; ^2^ Henan University of Engineering Zhengzhou China

**Keywords:** food processing industry, food security, industry growth, spatial shift‐share model

## Abstract

Faced with the pressure of slowing industrial growth and industrial transformation requirements, it is crucial to analyze the changes and the corresponding driving factors of the food processing industry in China. An analysis using traditional and spatial shift‐share models was conducted to decompose the changes in the food processing industry in each region of China from 2009 to 2019 into five effects: national growth effect (NG), industrial mix effect (IM), competitive effect (CE), neighbor‐nation competitive effect (NNC), and region‐neighbor competitive effect (RNC). Among the five effects from 2009 to 2019, the NG contributed the most to the growth in most regions, indicating that the development of the food processing industry in China was greatly influenced by the industrial base and that China's food processing industry has entered a “growth bottleneck period.” During the period 2009–2014 to period 2014–2019, compared to the IM and CE, the influence of spatial spillover effects was stronger and significantly enhanced. Moreover, the IM, CE, NNC, and RNC in most southern regions were stronger than those in most northern regions. Therefore, China's food processing industry needs and is transforming into high‐quality development. It is necessary to innovate the mode of development of food processing industry and strengthen interregional exchanges and cooperation.

## Graphical abstract

An analysis using traditional and spatial shift‐share models was conducted to decompose the changes in food processing industry in each region of China from 2009 to 2019 into five effects. This study found that from 2009 to 2019, the output value growth of the food processing industry in most regions of China was most affected by the industrial base, followed by the spatial spillover effect, and the least by the industrial structure.

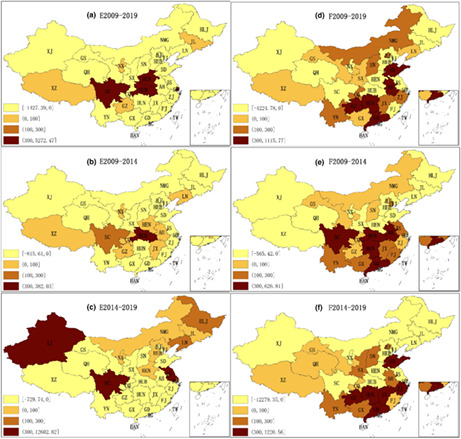

## 1 INTRODUCTION

Food security has long been a hot debating research topic globally (Cai et al., 2020; Godfray et al., 2010; Hanjra & Qureshi, 2010; Molotoks et al., 2021), which has been lifted to a higher significance on the policy agenda of many countries when facing international environment uncertainties, such as COVID‐19 and Russia–Ukraine conflict (Amare et al., 2021; Ben Hassen & El Bilali, 2022; Laborde et al., 2020, 2021). Ensuring food security means not only ensuring the efficiency and productivity of farms but also maintaining a strong processing capacity (Majiwa et al., 2018). As a key link of the food system, food processing industry plays a supporting role to ensure food security, and a driving role in the development of food system. Moreover, food processing industry is critical to meeting the diverse needs of consumers (Aguilar et al., 2019).

China is the world's largest food producer and consumer (Xu & Li, 2020). China produces one‐fourth of the world's food and it accounts for nearly one‐fifth of the world's population (State Council Information Office of the People's Republic of China, 2019). It is of great significance to focus on the development of China's food processing industry, as it closely associates with the livelihood of 1.4 billion people (Xie & Li, 2022). On the other hand, China has a large population yet a relatively small proportion of arable land. Food production and consumption have been in a tight balance for a long time. Based on China's agricultural resource endowment, dietary structure, and dietary habits, China's food security strategy is to ensure “the absolute security of staple foods and the basic self‐sufficiency of grains” (Niu et al., 2021; State Council Information Office of the People's Republic of China, 2019). Therefore, grain self‐sufficiency, grain cultivation, and grain processing are highly emphasized in China (Cao et al., 2021; Chen et al., 2011; Hang, 2021; Li, Sun, et al., 2021; Niu et al., 2022). Thus, this study focused on China's food processing industry mainly including flour, rice, oil, feed, and other cereal derivative food using data from “Statistics of Grain and Oil Processing Industry”.

In recent years, China's food processing industry has gained rapid development. With the continuous improvement of comprehensive grain production capacity, China's grain production has been abundant for years. The annual output has stabilized above 650 million tons (Li & Lv, 2021), laying a good foundation for further development of China's food processing industry. According to official data from the Food and Strategic Reserves Administration of China, the annual output value of China's food processing industry has remained above 3 trillion yuan since 2018. In 2019, the total industrial output value of China's food processing industry was 3.15 trillion yuan, which is up 170.39% from 2009. Its profit totaled 242.37 billion yuan, seven times more than that of 2009 when it was 31.20 billion yuan. For research and development, 10.74 billion yuan was invested four times more than that of 2.36 billion yuan in 2009. There are 3063 leading enterprises in industrialization, with a total industrial output value of 1.83 trillion yuan, an increasing 346.34% compared with 0.41 trillion yuan in 2009.

However, at the same time, the growth rate of China's food processing industry has decreased, with a growth rate of 130.09% from 2009 to 2014 while only 17.51% from 2014 to 2019. Moreover, in the past decade, with the increase in residents' income, Chinese consumers' demand for food is changing from “eat enough” to “eat healthy” (Li & Lv, 2021). In order to better meet the needs of consumers and to ensure food security, the Chinese government has guided China's food processing industry to produce food with better quality and to achieve a more sustainable development by improving resource efficiency (Cao et al., 2019; Gao et al., 2021; General Office of the State Council of China, 2017; Lu et al., 2022; Xie & Li, 2022; Xu & Li, 2020). It is in line with the transformation trend of the global food processing industry in recent years (Aguilar et al., 2019; FAO, 2019; Xu et al., 2020). Therefore, faced with the pressure of slowing industrial growth and industrial transformation requirements, it is crucial for Chinese policy‐makers to analyze the changes and the corresponding driving factors of food processing industry.

Shift‐share analysis was used to study the changes and the corresponding driving factors of food processing industry. Shift‐share analysis is a common structural competitive advantage analysis method, which examines influences of industrial structure, regional competitive differences, and spatial spillover effect on industrial growth (or economic growth) through the decomposition of industrial growth (or economic growth). This method helps to analyze the reasons for the differences in industrial growth in different regions, which broadened the research content of regional difference analysis (Dogru et al., 2020; Polyzos, 2019). Shift‐share analysis has been improved and a variety of models have been developed (Costantino et al., 2020; Lahr & Ferreira, 2020; Montanía et al., 2020). Among them, the traditional and the spatial shift‐share model are the two most popular models. The traditional shift‐share model was first proposed by Jones (1940) and Creamer (1942), then developed by Dunn (1960), and refined by Esteban (2000). The traditional shift‐share model is mainly used to analyze the contribution of industrial structure and regional competition differences to industrial growth (Li, Xing, et al., 2021). Spatial shift‐share model is mainly used to analyze the impact of spatial spillover effects (Espa et al., 2014). Nazara and Hewings (2004) considered the spatial interaction between regions, and first introduced the spatial shift‐share analysis. Based on the ideas of Nazara and Hewings (2004), Zaccomer (2006) constructed a complete spatial shift‐share model. Many scholars analyzed the changes in different industries in different regions using traditional or spatial shift‐share models (Capello & Cerisola, 2022; Dogru & Sirakaya‐Turk, 2017; Espa et al., 2014; Li, Xing, et al., 2021; Lv et al., 2021; Mayo & López, 2008). Studies have been carried out in many fields such as regional productivity (O'Leary & Webber, 2015), electricity consumption (Grossi & Mussini, 2018; Lin et al., 2019), tourism (Krabokoukis & Polyzos, 2020), and global rice export (Lakkakula et al., 2015). To the best of our knowledge, this might be the first study applying traditional and spatial shift‐share analysis to decompose China's food processing industry changes.

Until now, a number of studies have focused on the performance and development of food processing industry in China. Productivity and performance in the food processing industry is one of the research hotspots (Hockmann et al., 2018; Kapelko et al., 2015, 2016, 2017a, 2017b; Triguero et al., 2013). For example, Kapelko (2019) analyzed the productivity changes in food processing industry in EU countries, and Cardamone (2020) analyzed the productivity spillover effect among Italian food manufacturing enterprises. Regarding the related research on China's food processing industry, Dai et al. (2021) researched whether a higher market power would reduce efficiency based on the data from China's rice processing industry. On average, China's rice processing enterprises have relatively weak market power but face high‐cost efficiency. Fu et al. (2011) examined the technical efficiency of wheat and paddy rice processing in China during the period from 2005 to 2007. Xu and Li (2020) compared the performance of food processing and food manufacturing in China, and the results showed that the performance of food manufacturing enterprises was better than that of food processing enterprises. Sun et al. (2021) studied the spatial pattern of the Chinese green food industry and found that the structure and types of green food enterprises are relatively simple and the regional development is not unified. Furthermore, Zhang et al. (2018) explored the green development mode of the Chinese food industry and the total factor productivity of food processing industry based on the Industrial Internet of Things. Cao et al. (2019) investigated the sustainable development of Chinese food processing enterprises. These studies have provided insights into the understanding of the development and changes in the food processing industry in China. However, China is vast, and the food processing industry includes many subsectors. There is a lack of systematic analysis of the growth differences and the corresponding driving factors of food processing industry in different regions of China.

From the above discussion, we can infer that on the one hand, China's food processing industry is experiencing a decline in growth and is facing transformation pressure. Exploring the driving factors of the growth in food processing industry in each region and implementing a targeted development policy would definitely accelerate food processing industry growth. On the other hand, 2009–2019 is an important period for the transformation of the food processing industry in China, while changes in industrial structure reflect changes in consumer demand to a certain extent. Moreover, with the advances in information technology and the rapid development of logistics services in China, the cooperation and exchanges between regions have been strengthened. China's food processing industry changes in one region is inevitably influenced by its neighboring areas due to the existence of spatial spillovers. Therefore, both traditional and spatial models are used to analyze the effects of industrial structure and competitiveness as well as the spatial spillover effects. The output value growth of food processing industry in different regions of China from 2009 to 2019 has been decomposed into five components, which are national growth effect (NG), industrial mix effect (IM), competitive effect (CE), neighbor‐nation competitive effect (NNC), and region‐neighbor competitive effect (RNC). The contributions of these five components to the growth of food processing industry have been compared. Such analysis could help policy‐makers make targeted development policies. The practice of China's food processing industry over years is also helpful in promoting the development of food processing industry for other developing countries.

The rest of this research paper is arranged as follows: Section 2 is about models and data sources; the analysis and discussion of decomposition results are stated in Section 3; and research conclusions and implications are presented in Section 4.

## 2 METHODOLOGY AND DATA

The methodology for this study is conducting shift‐share analysis, and the statistical data of China's food processing industry from 2009 to 2019 is used. The output value growth of the food processing industry in 31 provincial administrative regions in China has been decomposed into five effects. ArcGIS software was applied to analyze and map the spatial and temporal patterns of the actual growth and the five effects.

### 2.1 Data sources and indicators

The data used for the shift‐share analysis are derived from the “Statistics of Grain and Oil Processing Industry” of the National Food and Strategic Reserves Administration of China. Since the latest data are not available, this study takes 2009 as the base period and 2019 as the end period to analyze the output value growth of the food processing industry in different regions. The data span 11 years, including 31 provincial‐level administrative regions (excluding Hong Kong, Macao, and Taiwan), which is representative to a certain extent. Some Chinese scholars have used the data from 2008 to 2014 of “Statistics of Grain and Oil Processing Industry” to study the total factor productivity and industrial agglomeration of food processing industry, and reached reliable conclusions (Chen & Zhong, 2017; Zhang, 2018). It can be seen that the data are applicable for industry analysis.

The division of periods is as follows, taking 2014 as the dividing point, so that it can take 5 years as a period. China's food processing industry has an industrial development plan from the government every 5 years. The period of the 12th Five‐Year Plan is 2010–2015, and the period of the 13th Five‐Year Plan is 2016–2020. Due to the lack of data for 2020, we have pushed the time forward by 1 year.

The division of industries is as follows. According to “Statistics of Grain and Oil Processing Industry,” China's food processing industry is subdivided into eight domains, including rice, flour, vegetable oil, corn, instant food, feed, coarse grains and deep processing, and grain machinery manufacturing. Coarse grains mainly refer to potatoes, beans, etc. Instant food includes instant noodles, instant rice porridge, frozen dumplings, rice dumplings, sweet dumplings, wine, etc. Changes in the feed processing industry indirectly reflect in an indirect way the changes in animal husbandry, meat, eggs, and dairy industries.

“Statistics of Grain and Oil Processing Industry” has been revised since 2015. The industrial subdivision of the statistical data changed, coarse grains were separated from deep processing, but corn was incorporated into the deep processing industry. To keep the same diameter, we have combined corn, coarse grains, and deep processing subsectors into one subsector, referred to as coarse grains. Thus, the food processing industry is subdivided into seven subsectors, including rice, flour, vegetable oil, instant food, coarse grains, feed, and grain machinery manufacturing.

The division of regions is as follows, with regions divided according to China's provincial administrative regions. There are 34 provincial‐level administrative regions in China. However, as some data from Hong Kong, Macao, and Taiwan are not included in the statistical data, these three provincial‐level administrative regions are not included in the research. The rest of the 31 provincial‐level administrative regions are Xinjiang (XJ), Ningxia (NX), Qinghai (QH), Gansu (GS), Shaanxi (SX), Xizang (XZ), Yunnan (YN), Sichuan (SC), Guizhou (GZ), Chongqing (CQ), Hainan (HAN), Guangxi (GX), Guangdong (GD), Jilin (JL), Hunan (HUN), Hubei (HUB), Henan (HEN), Shandong (SD), Jiangxi (JX), Fujian (FJ), Anhui (AH), Zhejiang (ZJ), Jiangsu (JS), Shanghai (SH), Heilongjiang (HLJ), Liaoning (LN), Nei Monggol (NMG), Shanxi (SN), Hebei (HEB), Tianjin (TJ), and Beijing (BJ). Furthermore, Hainan is an island and does not border other areas, so the effects of spatial shift‐share model are 0.

### 2.2 Shift‐share analysis

#### 2.2.1 Traditional shift‐share model

The traditional shift‐share analysis takes the whole country (or the region where it is located) as a reference and divides industrial growth into three parts, such as national growth effect (NG), industrial mix effect (IM), and competitive effect (CE). The variable used to measure industry growth can be output, production, employment, or other variables. This study investigates the industrial growth of the food processing industry in China. Since seven subdivision industries are involved, output value is adopted to measure the industrial growth in order to maintain consistency. The country's 31 provincial administrative regions are used as a reference.

The equation of the traditional shift‐share model is shown in Equation (1). In this equation, *i* represents the number of food processing subdivision industries, ranging from 1 to 7, where *m* = 7. *j* indicates the number of 31 provincial administrative regions, and its value ranges from 1 to 31. *r*_*ij*_ represents the output value growth rate of industry *i* among the food processing industry in region *j*. *R* represents the growth rate of the output value of the food processing industry in China. *R*_*i*_ is the national output growth rate of the *i* industry. *R*_*j*_ represents the growth rate of output value of food processing industry in j region. $r_{\mathrm{ij}}^{v}$ indicates the growth rate of the *i* food processing industry in the adjacent region of region *j*. $G_{j}^{t0}$ represents the base production value of the food processing industry in region *j*. $G_{j}^{t1}$ stands for the final output value of the food processing industry in region *j*, and $G_{\mathrm{ij}}^{t0}$ represents the base production value of industry *i* in region *j*.

(1) $$G_{j}^{t1}-G_{j}^{t0}=\sum_{i=1}^{m}G_{\mathrm{ij}}^{t0}r_{\mathrm{ij}}=\sum_{i=1}^{m}G_{\mathrm{ij}}^{t0}R+\sum_{i=1}^{m}G_{\mathrm{ij}}^{t0}\left(R_{i}-R\right)+\sum_{i=1}^{m}G_{\mathrm{ij}}^{t0}\left(r_{\mathrm{ij}}-R_{i}\right).$$

The first part on the right of the equation is the national growth effect (NG), which represents the amount increased by the food processing industry in region *j* according to the growth rate of the national food processing industry. The second part is the industrial mix effect (IM), which represents the sum of the amount increased by the subdivisions of the food processing industry in region *j* according to the difference between the growth rate of the output value of subdivisions and that of the whole industry in China. Its value is positive, indicating that most food processing subdivisions in this region have industrial growth advantages, and that this region has an industrial structure advantage. The third part is the competitive effect (CE), which represents the sum of the amount increased by the subdivisions of food processing industry in region *j* according to the difference between the growth rate of actual output value of the subdivisions in region *j* and that of the subdivisions in China. Its value is positive, indicating that the growth rates of most food processing subdivisions in region *j* are higher than that of other regions in the country and that this region has a competitive advantage.

#### 2.2.2 Spatial shift‐share model

Juxtapose with the traditional shift‐share model, the spatial shift‐share model considers the interaction between regions. Studies have shown that spatial spillover effects have an important impact on industrial development.

In this study, a 30 × 30 weight matrix *W* is constructed to represent the interaction among 30 provincial administrative regions. Since Hainan does not border other regions, a 30 × 30 weight matrix is constructed. *w*_*jk*_ is the spatial weight, representing the intensity of the interaction between regions *j* and *k*. Different definition standards of spatial weight will influence the results of empirical analysis to some extent. Therefore, the most classical (0–1) space–weight matrix construction method is selected. Geographically adjacent counts as 1, and not adjacent counts as 0. And the matrix *W* is normalized by row.

The calculation of the spatial shift‐share model is shown in Equation (2). The calculation of $r_{\mathrm{ij}}^{v}$ is shown in Equation (3), and other symbols are consistent with the symbolic meaning of Equation (1). In Equation (3), *v* is the number of adjacent regions of region *j*, and *w*_*jk*_ is the row‐normalized spatial weight defined earlier. $G_{\mathrm{ik}}^{t0}$ represents the base output value of the industry *i* in the adjacent regions of region *j*. $G_{\mathrm{ik}}^{t1}$ stands for the final output value of the industry *i* in the adjacent regions of region *j*.

(2) $$G_{j}^{t1}-G_{j}^{t0}=\sum_{i=1}^{m}G_{\mathrm{ij}}^{t0}r_{\mathrm{ij}}=\sum_{i=1}^{m}G_{\mathrm{ij}}^{t0}R+\sum_{i=1}^{m}G_{\mathrm{ij}}^{t0}\left(r_{\mathrm{ij}}^{v}-R\right)+\sum_{i=1}^{m}G_{\mathrm{ij}}^{t0}\left(r_{\mathrm{ij}}-r_{\mathrm{ij}}^{v}\right),$$

(3) $$r_{\mathrm{ij}}^{v}=\left(\sum_{k\in v}w_{\mathrm{jk}}G_{\mathrm{ik}}^{t1}-\sum_{k\in v}w_{\mathrm{jk}}G_{\mathrm{ik}}^{t0}\right)/\sum_{k\in v}w_{\mathrm{jk}}G_{\mathrm{ik}}^{t0}.$$

In Equation (2), the first part on the right is the national growth effect (NG), which is consistent with the national growth effect in Equation (1). The second part on the right side of the formula is the neighbor‐nation competitive effect (NNC), which represents the sum of the amount increased by the food processing subdivisions in region *j* according to the difference between the growth rate of the output value of the subdivisions in adjacent regions of region *j* and that of the whole industry in China. Its value is greater than 0, indicating that most of the food processing subdivision industries in adjacent regions of region *j* have industrial growth advantages, and that the region *j* has a spatial structure advantage. The third part is the region‐neighbor competitive effect (RNC), which indicates the sum of the amount increased by the food processing subdivisions in region *j* according to the difference between the growth rate of actual output value of the subdivisions in region *j* and that of the subdivisions in the neighboring regions of region *j*. Its value is greater than 0, indicating that the growth rates of most food processing subdivisions in region *j* are higher than that of the industry in neighboring regions, and that the region *j* has a spatial competitiveness advantage.

## 3 RESULTS AND DISCUSSION

### 3.1 Growth differences of food processing industry and its subdivisions in China from 2009 to 2019

From 2009 to 2019, the production value of China's food processing industry kept rising, showing a stable and high‐speed development trend on the whole, but the growth rate declined. In 2019, the total industrial production value of China's food processing industry was 3.15 trillion yuan, up 170.39% from 2009. However, the growth rate of the food processing industry has slowed down, with the industry growing by 130.09% from 2009 to 2014 and only 17.51% from 2014 to 2019.

According to Table 1, the seven subdivision industries of the food processing industry all showed positive growth from 2009 to 2019. From 2009 to 2014, the growth rate of all subsectors was fast. From 2014 to 2019, the growth rate of food processing subsectors declined significantly, while rice, flour, and vegetable oil industries showed negative growth.

**TABLE 1:** Growth rate of production value of food processing industry and its subdivisions in China (%)

Time period	Rice (%)	Flour (%)	Vegetable oil (%)	Instant food (%)	Coarse grains (%)	Feed (%)	Grain machinery (%)	Food processing industry (%)
2009–2019	147.77	106.15	53.41	1030.14	87.80	280.85	199.39	170.39
2009–2014	156.21	118.76	70.42	326.59	57.99	237.33	191.91	130.09
2014–2019	−3.30	−5.76	−9.98	164.93	18.87	12.90	2.56	17.51

From 2009 to 2019, the growth rate of instant food, feed, and grain machinery was fast, and the growth rate of output value was higher than the average growth rate of the food processing industry. Among them, instant food was growing the fastest and led by absolute advantage. The feed growth rate of 280.85%, ranked second. Grain machine increased by 199.39%, ranking third. The rapid growth of the feed industry is related to the changes in China's diet structure in recent years (Niu et al., 2021). As China's national strength improves and people's income increases, the consumption demand for meat products continues to grow.

From 2009 to 2014, the production value of instant food, feed, grain machinery, and rice grew faster than the average growth rate of the food processing industry. Among them, instant food processing had the fastest growth rate and the growth rate of production value was 326.59%. Vegetable oil grew the slowest, with a growth rate of 70.42%.

From 2014 to 2019, the growth rate of production value of the food processing industry and its subsectors declined significantly, when compared with that of 2009–2014. The growth rate of instant food, coarse grain, and feed industries was fast, and the growth rate of the coarse grain industry ranked second, and while rice, flour, and vegetable oil showed negative growth.

### 3.2 Actual growth and national growth effect of food processing industry by region

**FIGURE 1:**
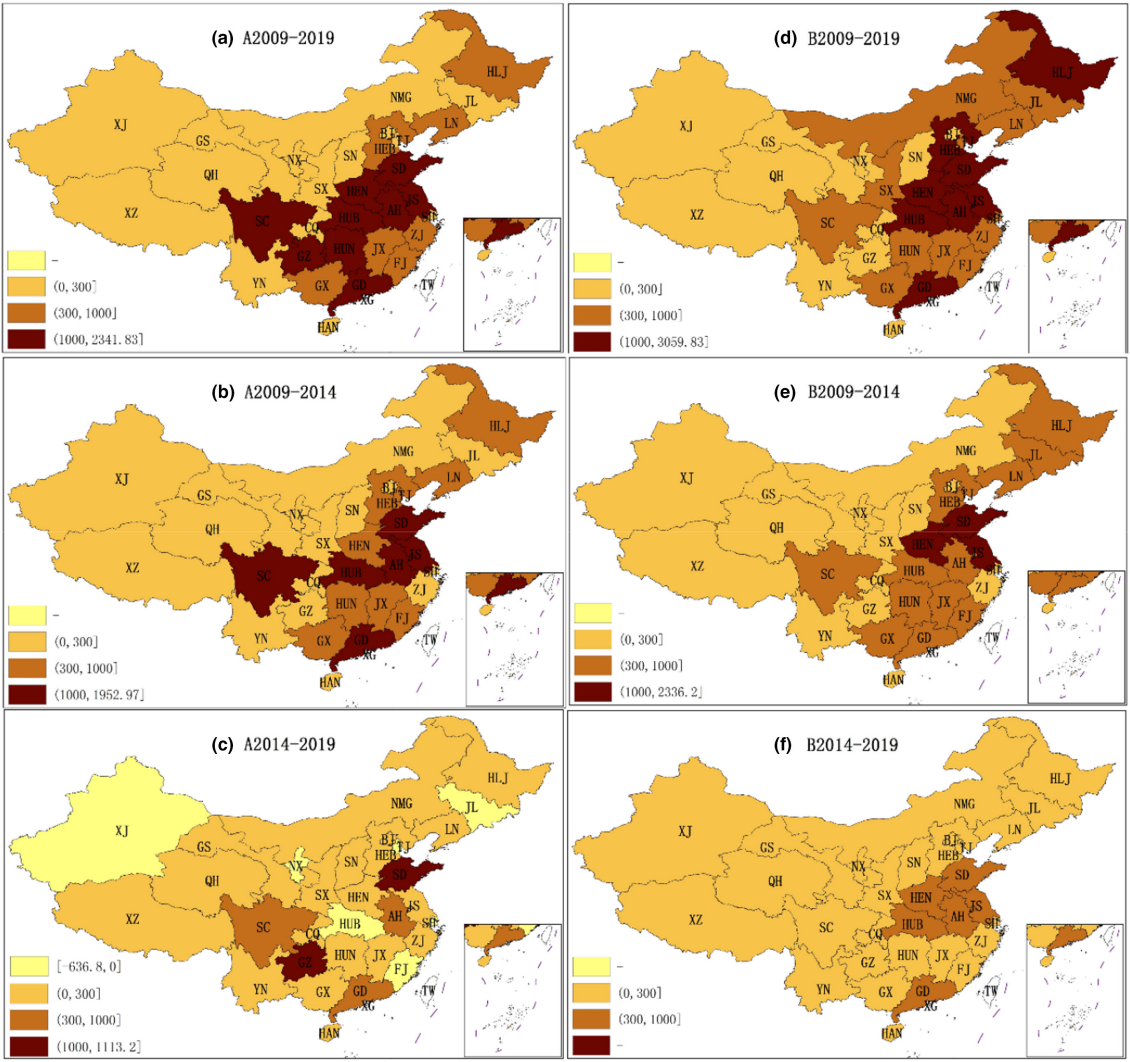
Spatiotemporal variation in actual growth and national growth effect (unit: 100 million yuan)

Figure 1a–c reflect the actual growth of the food processing industry in different regions during different periods. The period from 2009 to 2014 was similar to the period from 2009 to 2019, with the food processing industry showing positive growth in all regions. From 2014 to 2019, the food processing industry in most regions showed positive growth, but the growth quota of most regions was lower than the growth quota of 2009–2014. For example, TJ, JL, SH, FJ, HUB, NX, and XJ even showed negative growth.

Figure 1d–f indicate the national growth effect in the food processing industry in different regions during different periods. The national growth effect (NG) reflects the industrial base of the food processing industry in each region and the contribution of the industrial base to the regional industrial growth. According to Figure 1, the food processing industry in eastern and central China has a relatively good industrial base.

### 3.3 The result analysis of the traditional shift‐share model

**FIGURE 2:**
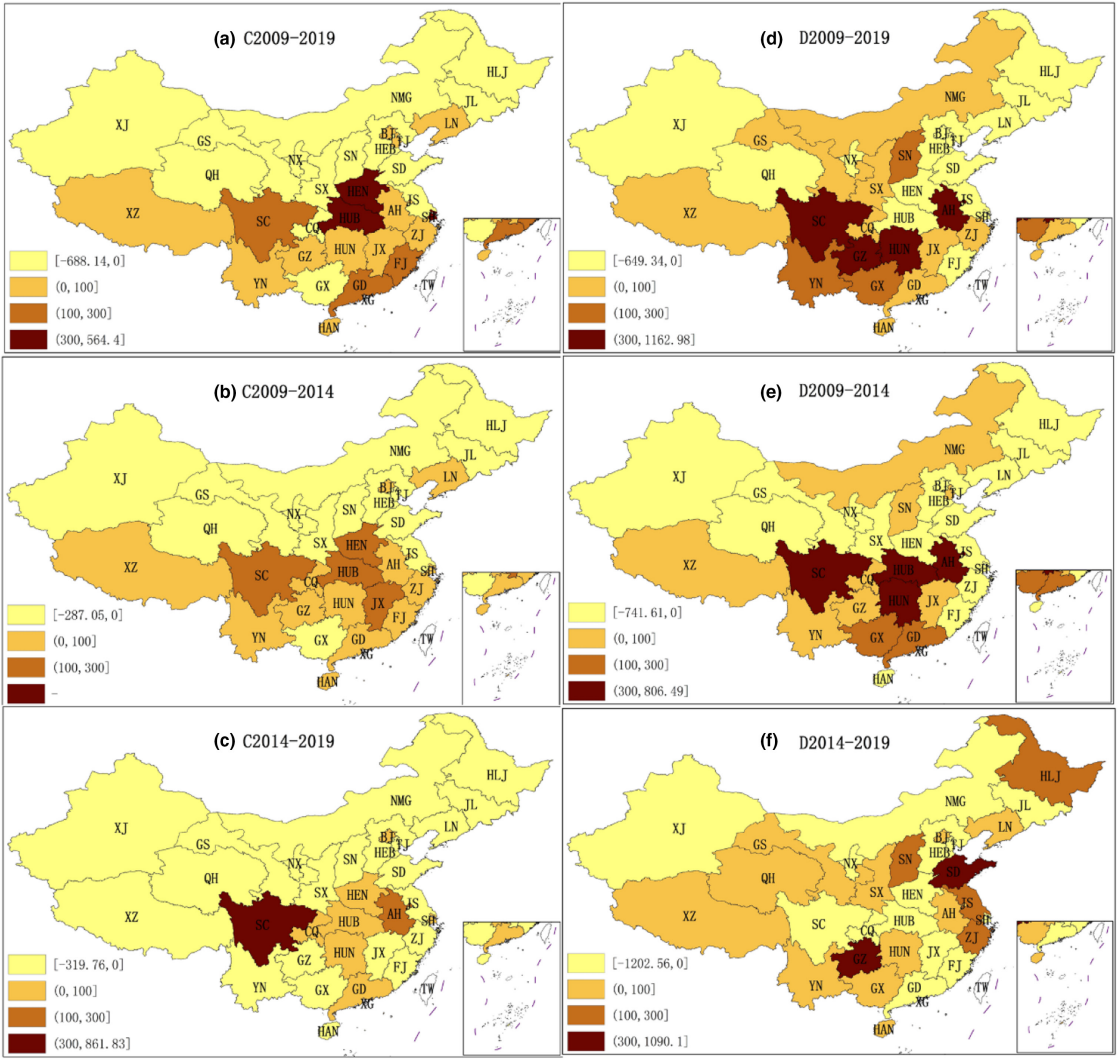
Spatiotemporal variation in industrial mix effect and competitive effect (unit: 100 million yuan)

According to the traditional shift‐share Model, the industrial mix effect (IM) and competitive effect (CE) of China's food processing industry from 2009 to 2019, 2009 to 2014, and 2014 to 2019 are calculated, respectively. Figure 2a–c reflect the spatiotemporal change in industrial mix effect. And Figure 2d–f reflect the spatiotemporal change in competitive effect.

#### 3.3.1 The spatiotemporal change in industrial mix effect

The industrial mix effect (IM) reflects the rationality of industrial structure layout in this region. A positive IM means the region has advantage of industrial structure. The larger the value, the more reasonable is the regional structure layout.

According to Figure 2a, from 2009 to 2019, the south had more industrial structure advantages than the north. The industrial mix effect of most provincial administrative regions in North China is negative except for LN, BJ, and TJ. The industrial mix effect of most provincial administrative regions in South China is positive, except for GX. Among them, HUB province had the highest IM of 56.440 billion yuan. HEN ranked second, at 49.712 billion yuan.

According to Figure 2b, the situation during the 2009–2014 period was similar to the 2009–2019 period. The industrial mix effect of most provincial administrative regions in North China is negative except for LN and BJ. The industrial mix effect of most provincial administrative regions in South China is positive. There were 13 regions with an IM of between 0 and 10 billion yuan and four regions with an IM of between 10 and 30 billion yuan. Among which, HUB's IM was 20.235 billion yuan, ranking first. The IM of JX was 14.754 billion yuan, ranking second. SC's IM was 11.649 billion yuan, ranking third. HEN's IM was 11.142 billion yuan, ranking fourth.

According to Figure 2c, the industrial mix effect in most regions was negative from 2014 to 2019. Among the 31 provincial‐level administrative regions, only eight provincial‐level administrative regions had a positive industrial mix effect. SC's IM was 86.183 billion yuan, ranking first. AH's IM was 12.265 billion yuan, ranking second. HUB ranked third with an IM of 9.664 billion yuan. Furthermore, SC had the highest industrial mix effects because of its food industrial structure adjustment. Such adjustment increased the distribution of the instant food processing industry and resulted in the highest proportion of the instant food processing industry. Moreover, the main increase was in the brewing industry.

#### 3.3.2 The spatiotemporal change in competitive effect

The larger the competitive effect, the stronger the competitiveness of most food processing subsectors in this region.

According to Figure 2d, from 2009 to 2019, the competitive effect of 15 provincial‐level administrative regions was negative, while that of 16 provincial‐level administrative regions was positive. The central and southern regions were more competitive than the northern industries. There were nine provincial‐level administrative regions with a CE of 0–10 billion yuan and three regions with a competitive effect of 10–30 billion yuan. There were four provincial‐level administrative regions with a CE of more than 30 billion yuan, among which GZ's CE was 116.298 billion yuan ranking first. The CE of SC was 81.263 billion yuan, ranking second. AH's CE was 75.622 billion yuan, ranking third. HUN's CE was 64.645 billion yuan, ranking fourth. Furthermore, the industrial competitiveness of GZ and SC was more prominent. The main reason is that from 2009 to 2019, the liquor industry in these two regions, belonging to the instant food processing industry, was developing relatively fast, especially in the latter stage with outstanding production value growth, as represented by “Kweichow Moutai.”

According to Figure 2e, from 2009 to 2014, the competitive effect of 17 provincial administrative regions was negative, while the competitive effect of 14 regions was positive. There were eight regions with a CE of between 0 and 10 billion yuan and two regions with a CE of between 10 and 30 billion yuan. There were four provincial administrative regions with more than 30 billion yuan, among which, HUB's CE was 80.649 billion yuan, ranking first. AH's CE was 52.471 billion yuan, ranking second. The CE of SC was 49.979 billion yuan, ranking third, and HUN's CE was 47.386 billion yuan, ranking fourth.

According to Figure 2f, from 2014 to 2019, the competitive effect of 14 provincial‐level administrative regions was negative, while the competitive effect of 17 regions was positive. There were 11 provincial‐level administrative regions with a CE of 0–10 billion yuan and four provincial‐level administrative regions with a CE of 10–30 billion yuan. There were two regions with more than 30 billion yuan, among which, GZ's CE was 109.10 billion yuan, ranking first. SD's CE was 81.036 billion yuan, ranking second. The reason for GZ ranking first was mainly because its liquor industry grew rapidly, while other food processing industry performance was mediocre. Most subdivisions of food processing industry in SD grew faster and developed more evenly.

On the whole, the distribution of competitive effect from 2009 to 2014 was similar to that from 2009 to 2019. Besides, the distribution of competitive effect from 2014 to 2019 changed greatly compared with the previous stage. Moreover, HUB, AH, SC, and HUN were the most competitive provincial‐level administrative regions from 2009 to 2014, while GZ and SD were the most competitive regions from 2014 to 2019.

### 3.4 Results analysis of spatial shift‐share model

**FIGURE 3:**
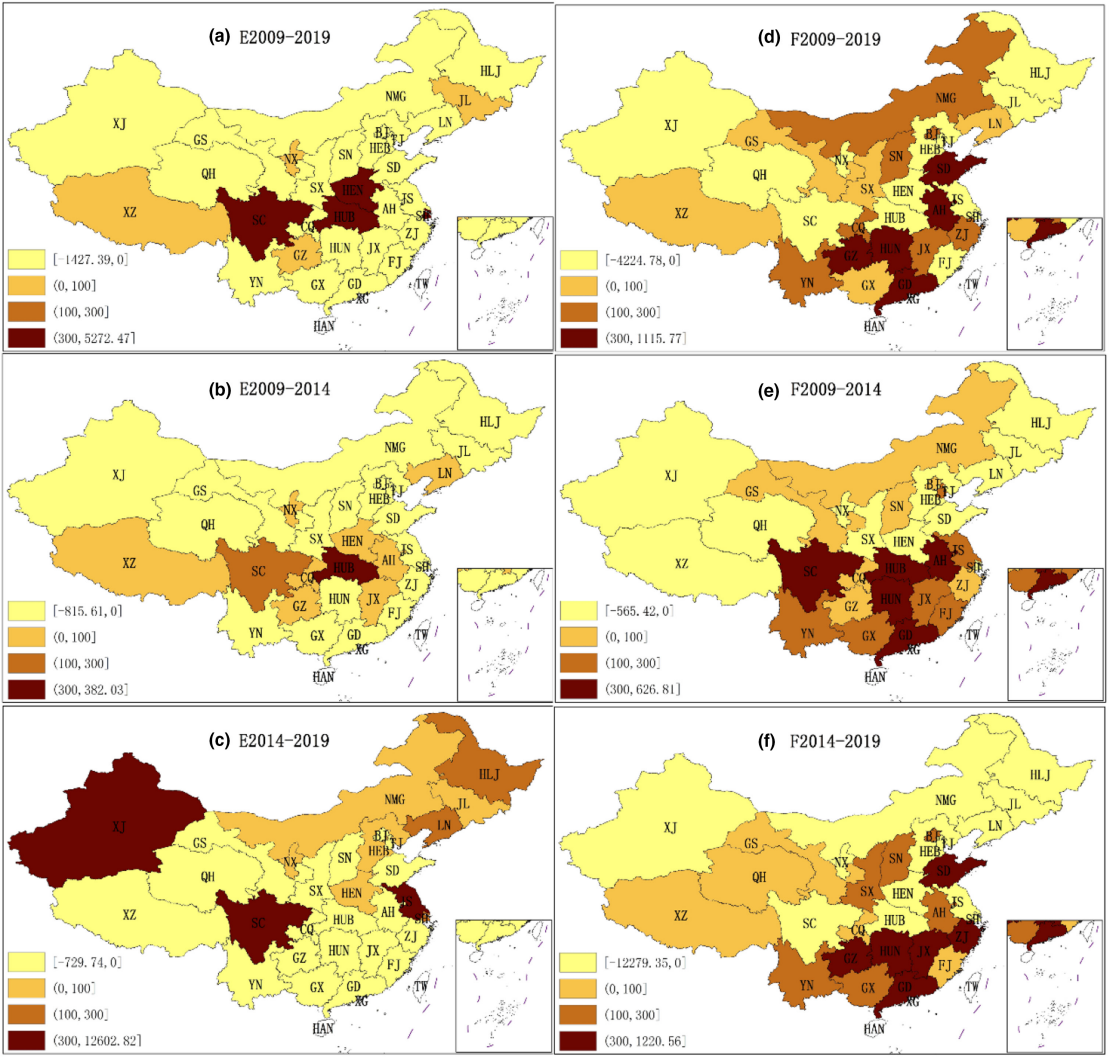
Spatiotemporal changes in neighbor‐nation competitive effect and region‐neighbor competitive effect (unit: 100 million yuan)

Based on spatial shift‐share model, the neighbor‐nation competitive effect and region‐neighbor competitive effect of China's food processing industry from 2009 to 2019, 2009 to 2014, and 2014 to 2019 were calculated, respectively. Figure 3a–c, reflect the spatiotemporal changes in neighbor‐nation competitive effect. And Figure 3d–f reflect the spatiotemporal changes in region‐neighbor competitive effect. Since HAN is an island and does not border other provincial administrative regions, the result of the effects of spatial shift‐share model of Hainan is 0.

#### 3.4.1 Spatiotemporal change in neighbor‐nation competitive effect

A positive neighbor‐nation competitive effect reflects the rationalization of industrial structure in the adjacent area. And the industrial structure of the adjacent area would have a positive impact on it. The bigger the value, the greater the influence of the neighboring region.

According to Figure 3a, from 2009 to 2019, the neighbor‐nation competitive effect of most provincial administrative regions was negative, while that of eight regions was positive. There were four regions with a NNC of 0–10 billion yuan and four regions with a NNC of more than 30 billion yuan. Among them, the SC's NNC was 527.247 billion yuan, ranking first. HUB's NNC was 71.809 billion yuan, ranking second. SH's NNC was 35.802 billion yuan, ranking third and HEN's NNC was 32.93 billion yuan, ranking fourth.

According to Figure 3b, from 2009 to 2014, the neighbor‐nation competitive effect of most provincial administrative regions was negative, while that of 10 regions was positive, and most of the positive regions were located in the south. Among them, there were eight regions with a NNC between 0 and 10 billion yuan, and one region with a NNC between 10 and 30 billion yuan. One region had a NNC over 30 billion yuan. HUB's NNC was 38.203 billion yuan, ranking first, and SC's NNC was 13.115 billion yuan, ranking second.

According to Figure 3c, from 2014 to 2019, the neighbor‐nation competitive effect of most provincial administrative regions in South China was negative, while that of 11 regions was positive. Among them, there were five regions with a NNC between 0 and 10 billion yuan, and three regions with a NNC between 10 and 30 billion yuan. Three regions had more than 30 billion yuan of NNC, among which, SC's NNC was 1260.282 billion yuan, ranking first. XJ's NNC was 98.255 billion yuan, ranking second, and JS's NNC was 37.172 billion yuan, ranking third.

On the whole, the distribution of neighbor‐nation competitive effect in 2009–2014 was similar to that in 2009–2019, and there was a big difference between the period 2014–2019 and the previous stage. It indicated that the initial share of the food processing industry had great influence. From 2009 to 2014, most of the provinces with positive neighbor‐nation competitive effects belonged to the south, while HUB province had a strong neighbor‐nation competitive effect. From 2014 to 2019, most of the regions with positive neighbor‐nation competitive effect belonged to northern China, where SC, XJ, and JS had strong neighbor‐nation competitive effects.

#### 3.4.2 Spatiotemporal change in region‐neighbor competitive effect

A positive region‐neighbor competitive effect indicates that the growth of most food industries in this region is better than that in its adjacent regions, with spatial competitiveness advantages and spatial spillover effects.

According to Figure 3d, from 2009 to 2019, the region‐neighbor competitive effect of 13 provincial‐level administrative regions was negative, while that of 17 regions was positive. Among them, there were five regions with a RNC of between 0 and 10 billion yuan and seven regions with a RNC of between 10 and 30 billion yuan. There were five regions and the regions shared more than 30 billion yuan of spatial competitiveness, among which, GZ's RNC was 111.577 billion yuan, ranking first. AH's RNC was 96.901 billion yuan, ranking second with HUN's RNC of 86.636 billion yuan, ranking third. SD's RNC was 70.939 billion yuan, ranking fourth, and GD's RNC was 44.687 billion yuan, ranking fifth.

According to Figure 3e, from 2009 to 2014, the region‐neighbor competitive effect of 12 provincial‐level administrative regions was negative, while that of 18 regions was positive. Among them, there were seven regions with a RNC of between 0 and 10 billion yuan and six regions with a RNC between 10 and 30 billion yuan. There were five regions with a RNC of more than 30 billion yuan, among which HUB province ranked first with a RNC of 62.681 billion yuan. Secondly, HUN's RNC was 61.64 billion yuan, ranking second. Thirdly, AH's RNC was 57.66 billion yuan ranking third, SC's RNC at 48.514 billion yuan ranking fourth, and lastly, GD's RNC was 42.947 billion yuan ranking fifth.

According to Figure 3f, from 2014 to 2019, the region‐neighbor competitive effect of 13 provincial‐level administrative regions was negative, while that of 17 regions was positive. Among them, there were five regions with a RNC of between 0 and 10 billion yuan and six regions with a RNC of between 10 and 30 billion yuan. There were six regions with an RNC of more than 30 billion yuan, among which SD ranked first with an RNC of 122.056 billion yuan. GZ's RNC was 113.668 billion yuan, ranking second and GD's RNC was at 69.92 billion yuan, ranking third. Furthermore, HUN's RNC was 49.041 billion yuan ranking fourth, and lastly, JX's RNC was 38.037 billion yuan, ranking fifth.

On the whole, the distribution of region‐neighbor competitive effect in 2009–2014 was similar to that in 2009–2019, and there was a big difference between the period 2014–2019 and the previous stage. It indicated that the initial share of the food processing industry had great influence. From 2009 to 2014 and from 2014 to 2019, the RNC in southern China was stronger than that in northern China. Lastly, from 2009 to 2014, HUB, HUN, AH, SC, and GD had a strong region‐neighbor competitive effect. From 2014 to 2019, SD, GZ, GD, HUN, and JX had a strong region‐neighbor competitive effect.

In general, from 2009 to 2019, the IM, CE, NNC, and RNC in the south were stronger than those in the north. The situation was similar during the 2009–2014 period. From 2014 to 2019, other things remained the same, the northern regions had better neighbor‐nation competitive effects than the southern regions. On the whole, the food processing industry in the south developed better in the past 10 years. This may be related to the better economic development in South China. Moreover, in fact, South China had a smaller advantage in terms of raw grain cultivation. The raw grain output in North China was higher than that in the South, and the grain logistics obviously showed a trend of “grains being transported from north to south.”

### 3.5 Comparison between different effects

**FIGURE 4:**
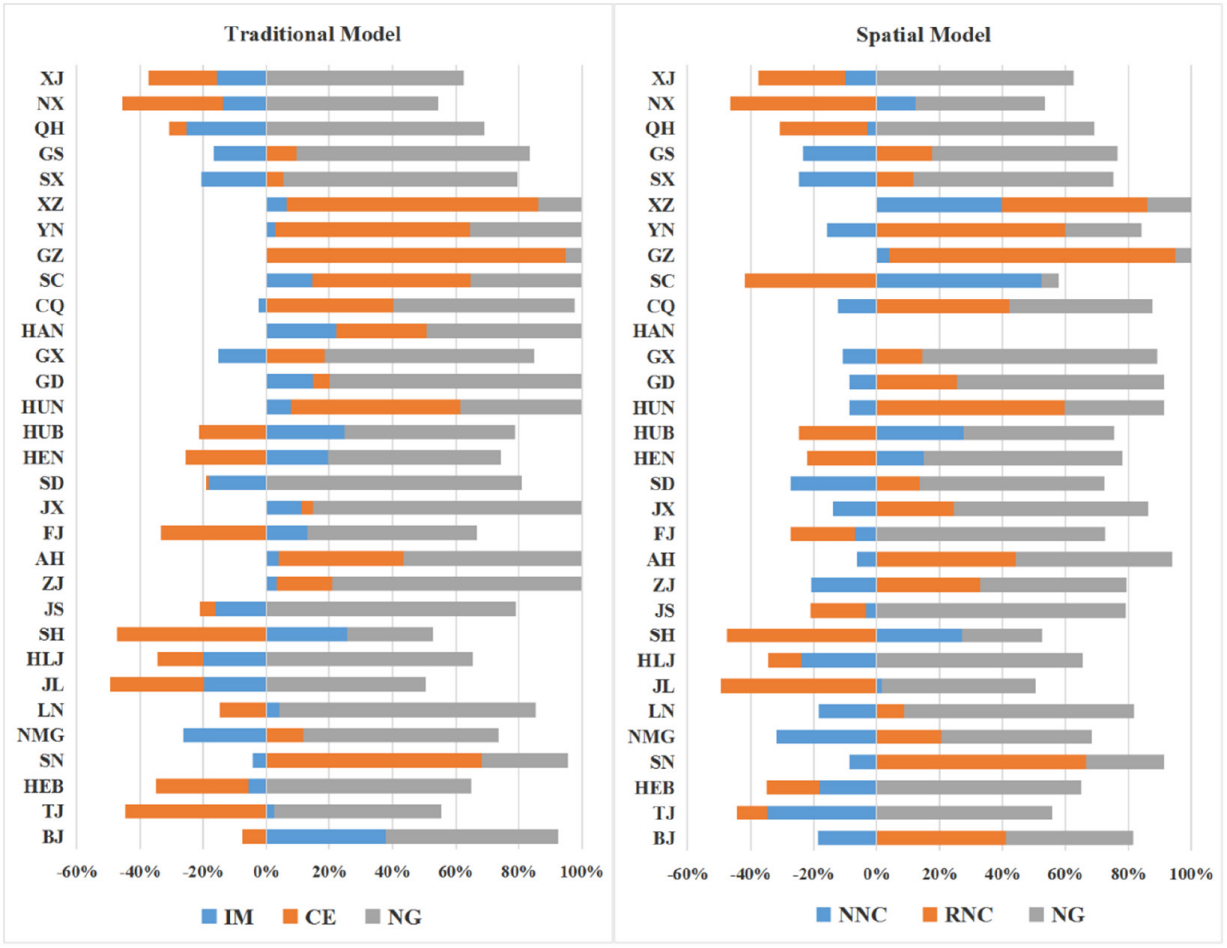
Comparison of different effects proportions from 2009 to 2019

Firstly, among the five effects of output value growth decomposition from 2009 to 2019, the national growth effect contributed the most, indicating that China's food processing industry has entered a “growth bottleneck period.” Table 2 shows the sum of the absolute value of regions with the five effects. According to Table 2, the sum of the absolute value of the national growth effect was 1957.776 billion yuan, which was the largest. The sum of the absolute value of neighbor‐nation competitive effect and region‐neighbor competitive effect ranked second and, on the other hand, the sum of the absolute value of industrial mix effect and competitive effect was the smallest. Figure 4 shows the proportions of different effects in China's 31 provincial‐level administrative regions from 2009 to 2019. According to Figure 4, from 2009 to 2019, whether compared with the effects of traditional model or the effects of spatial model, the national growth effect made the largest contribution to industrial growth in most regions of China. This showed that the development of the food processing industry in China was greatly influenced by the industrial base over the past 10 years. The spatial spillover effects ranked second, and the impact of industrial structure and competitiveness was the least. Moreover, with the slowdown of the growth rate of the food processing industry, it indicated that China's food processing industry entered a period of stagnation. This finding corroborates the findings of Xu and Li (2020) that the performance of food processing enterprises showed a downward trend according to the data of listed companies in China from 2016 to 2018. Therefore, China's food processing industry needs transformation. This result matches the findings of Cheng and Wang (2017).

**TABLE 2:** Sum of the absolute value of regions with the five effects (unit: billion yuan)

Time period	National growth effect	Industrial mix effect	Competitive effect	Neighbor‐nation competitive effect	Region‐neighbor competitive effect
2009–2019	1905.776	474.899	841.029	1086.889	1392.530
2009–2014	1455.068	213.737	583.561	391.301	547.905
2014–2019	450.705	259.597	585.284	1875.561	2176.620

*Note*: The value in the table is the absolute value sum of all regions of the effect during the period.

In addition, the five effects' contributions to the growth of the food processing industry in each region from 2009 to 2019 could be compared according to Figure 4. For instance, the competitiveness of the food processing industry in BJ from 2009 to 2019 was weak, but better than its adjacent regions. And the food processing industry in BJ has an excellent industrial structure simultaneously. In contrast to BJ, the food processing industry in SC had advantages of industrial structure and competitiveness from 2009 to 2019 but was weaker than its adjacent regions. And the industrial structure of its adjacent areas had a great positive impact on it.

**FIGURE 5:**
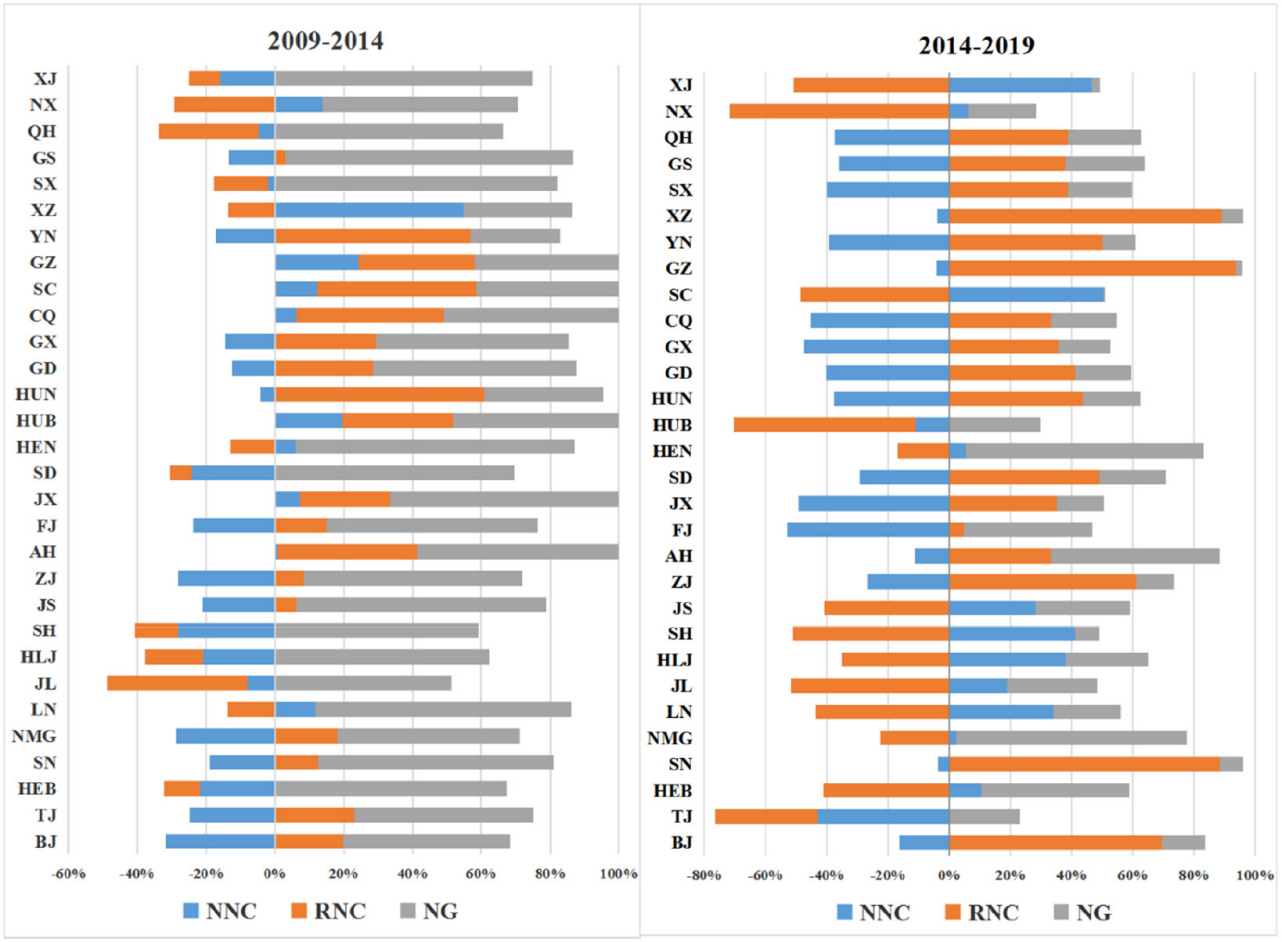
Two periods comparison of the effects proportions of spatial model

Secondly, compared with the two periods from 2009 to 2014 and from 2014 to 2019, the industrial mix effect and competitive effect did not change much, while the impact of neighbor‐nation competitive effect and region‐neighbor competitive effect increased significantly. According to Table 2, the sum of the regional absolute value of industrial mix effect and competitive effect did not change much in these two periods. The regional absolute summation of neighbor‐nation competitive effect increased from 391.301 billion yuan to 1875.561 billion yuan, and the regional absolute summation of region‐neighbor competitive effect increased from 547.905 billion yuan to 2176.62 billion yuan, thus, indicating a significant increase in spatial effects. Figure 5 shows the percentage of the effects of Spatial model for each region in both periods. According to Figure 5, for most regions, the influence of neighbor‐nation competitive effect and region‐neighbor competitive effect enhanced, no matter whether it was positive or negative. According to the data from these two periods, the spatial spillover effects increased significantly. Therefore, it can be inferred that promoting interregional resource flow and cooperation will be more conducive to the development of China's food processing industry.

## 4 CONCLUSIONS

In recent years, the growth rate of China's food processing industry has slowed down, and the Chinese government is guiding China's food processing industry to transform into high‐quality development mode. However, most of the related research is qualitative analysis, lacking the support of empirical analysis. This study provides empirical support for the formulation of relevant policies in China's food processing industry. The main conclusions are as follows. Firstly, China's food processing industry maintained a high growth rate from 2009 to 2019, but its growth rate declined significantly from 2014 to 2019. Secondly, from 2009 to 2019, the regional growth rates of the food processing industry and its subsectors varied in different regions, but basically, all showed positive growth, while some regions showed negative growth from 2014 to 2019. Thirdly, in terms of industrial structure, instant food, feed, and grain machinery manufacturing showed better growth advantages compared with other food processing subsectors. Interestingly, traditional industries such as rice, flour, and vegetable oil processing saw negative growth from 2014 to 2019. Fourthly, from 2009 to 2019, the IM, CE, NNC, and RNC in most southern regions were stronger than those in most northern regions of China. The situation was similar during the 2009–2014 period. However, from 2014 to 2019, other things remained the same, and most of the northern regions had better NNC than the southern regions. On the whole, the food processing industry in the south developed better in the past 10 years. Fifthly, by comparing the five effects, the national growth effect contributed the most to the growth of the food processing industry from 2009 to 2019. It illustrated that the initial share had the greatest impact on the growth of the food processing industry, while other factors had a relatively small impact on the industrial growth, indicating that food processing entered a period of stagnation. Lastly, during the period 2009–2014 to period 2014–2019, compared to the IM and CE, the influence of spatial spillover effects was stronger and significantly enhanced.

The implications of this study are as follows. Firstly, with the increase in people's income and the improvement in living standards, the diet structure of Chinese people has become diversified. Therefore, the intake of meat, seafood, soy products, vegetables, fruits, and nuts has increased, while the intake of staple foods such as rice and flour products has decreased. The demand for green and high‐quality food products increased, while the demand for ordinary food products decreased. For China's food processing industry, it is both a challenge and an opportunity. Secondly, China's food processing industry has slowed down in growth and entered a “growth bottleneck period,” which requires transformation to high‐quality development. It is very necessary to find a new growth point, transform development power, and innovate the development mode for China's food processing industry. This paradigm shift could promote the transformation of the food processing industry to high‐quality development. For example, the transformation and upgrading of the food processing industry can be promoted by improving the supply capacity of high‐quality food products, the utilization efficiency and comprehensive benefits of resources, and the green and sustainable development capacity. Thirdly, it is suggested that exchange and cooperation among regions should be strengthened. Thus, the influence of industrial mix effect is relatively small, and the influence of spatial effects is gradually strengthened, indicating that enhancing regional cooperation is more conducive to the development of the food processing industry.

Some limitations of the study are as follows. Firstly, this study only considered the impact of industrial structure, competitiveness, and adjacent area on the growth of the food processing industry, without considering the impact of other factors such as industrial policies and technological input. Besides, although traditional and spatial shift‐share models are used to analyze the growth and change in the food processing industry, further causal analysis is lacking, and the causal relationship needs more rigorous exploration. These deficiencies can be improved in future research.

## CONFLICT OF INTEREST

The authors declare no conflict of interest.

## Data Availability

The data that support the findings of this study are available from the National Food and Strategic Reserves Administration of China. Restrictions apply to the availability of these data, which were used under license for this study. Data are available from the authors with the permission of the National Food and Strategic Reserves Administration of China.
